# Sleep and happiness: socio-economic, population and cultural correlates of sleep duration and subjective well-being in 52 countries

**DOI:** 10.3389/frsle.2023.1118384

**Published:** 2023-07-26

**Authors:** Timo Lajunen, Esma Gaygısız, Wei Wang

**Affiliations:** ^1^Department of Psychology, Norwegian University of Science and Technology (NTNU), Trondheim, Norway; ^2^Department of Economics, Middle East Technical University, Ankara, Türkiye

**Keywords:** sleep duration, happiness, subjective well-being, cultural differences, socio-economic, population, Hofstede, individualism

## Abstract

**Introduction:**

Getting enough sleep is one of the essential lifestyle factors influencing health and well-being. However, there are considerable differences between countries in how much people sleep on average. The present study investigated how socio-economic factors, population variables, and cultural value dimensions are related to sleep duration in a sample of 52 countries.

**Method:**

The study design was ecological, i.e., the aggregate values for each country were obtained, and their correlations to national average sleep duration were analysed. The sleep duration estimates were based on Sleep Cycle Application (Sleep Cycle AB, Gothenburg, Sweden) data. The socio-economic variables included the economic health of a country (GDP per capita), how well a country is governed (governance quality measured with WGI), and the economic inequality (the gap between rich and poor measured with the Gini index) within a nation. The population variables included the urbanisation rate (proportion of people living in urbanised areas), life expectancy at birth, mean years of schooling among the population aged 25 years and older, median age of the population, and the prevalence of obesity (% of adults with BMI ≥ 30). The cultural value dimensions were measured with Hofstede's cultural value dimensions (power distance, individualism, masculinity, uncertainty avoidance, long-term orientation, and indulgence). The data were analysed by using zero-order correlations, partial correlations, and canonical correlation analyses.

**Results:**

Results showed a relatively strong intercorrelation between the national average of sleep duration and national happiness, i.e., subjective well-being. Among the socio-economic variables, WGI had the strongest relationship to sleep, whereas among population variables, schooling and obesity had the strongest correlations with sleep. Zero-order correlations between sleep and power distance and individualism were statistically significant, whereas in the partial correlations, individualism and masculinity appeared as important factors. Canonical correlation analysis showed strong correlations between the well-being variables (sleep and happiness) and the socio-economic variables, well-being variables and population variables, and cultural values and well-being variables.

**Discussion:**

The present study is an opening for a new line of research in which sleep is seen as an essential part of societal life and collective well-being.

## 1. Introduction

Sleep duration is widely recognized as one of the essential lifestyle contributors to health and well-being (St-Onge et al., 2016). Earlier studies have shown that short sleep duration increases the risk of cardiovascular disease and coronary heart disease (St-Onge et al., 2016; Li et al., 2022), cancer, metabolic syndrome, and stroke (Lee et al., 2015; Sivertsen et al., 2021; Li et al., 2022). Also, short sleep duration has been related to cognitive decline (Li et al., 2022) and poor mental health (Vaingankar et al., 2020; Braçe et al., 2022) such as depression (Lee et al., 2015; Stickley et al., 2019; Chen et al., 2020; Xiang et al., 2021), stress (Lee et al., 2015), and anxiety (Xiang et al., 2021). These findings show that short sleep duration effects negatively various aspects of life.

In addition to above mentioned various clinical outcomes, short sleep duration and low sleep quality can be expected to be related to people's life quality in general. This can be seen in people's evaluations of their happiness score, i.e., subjective well-being (SWB), sometimes also called as Cantril life ladder (Helliwell et al., 2022). In SWB, the respondents are asked to imagine a ladder, with steps numbered from zero to ten, in which the bottom (score 0) represents the worst possible life and the top (score 10) the best possible life. Hence, the ladder score reflects people's overall happiness level or SWB. In earlier studies, low sleep quality or duration has been negatively related to self-evaluated happiness, well-being or life satisfaction (Shin and Kim, 2018; Zhao et al., 2019; Otsuka et al., 2020; Kouros et al., 2022; Kukade et al., 2022). Good night's sleep is not related only to physical health but also to general life satisfaction and well-being.

While not getting enough sleep seems to be a global problem, there are considerable regional differences in sleep duration both among adults and adolescents (Gradisar et al., 2011; Matricciani et al., 2012; Bin et al., 2013; Gildner et al., 2014; Tozer, 2018). Earlier research shows that sleep duration differs across countries and cultures, with people in low- and middle-income countries getting less sleep than people in high-income countries (Simonelli et al., 2018). Regional differences in sleep duration can be expected because sleep duration and quality are known to be affected by cultural, social, and environmental influences, which vary significantly between regions (Schokman et al., 2018; Simonelli et al., 2018). In addition to geographical factors influencing sleep (Brockmann et al., 2017), cultural factors can be broken down into three types of variables: socio-economic factors, population characteristics and cultural values.

In the present study, the economic situation in a country was measured with income level (GDP per capita), income inequality (Gini index) and quality of governance with Worldwide Governance Indicators (WGI) published by the World Bank (World Bank, 2020). WGI consists of the following six dimensions of governance: Voice and Accountability, Political Stability and Absence of Violence, Government Effectiveness, Regulatory Quality, Rule of Law, and Control of Corruption. These six aggregate indicators combine the views of many enterprises, citizens, and experts. The WGI indexes are not meant to be used as distinct measures of different aspects of governance, but each of the indexes reflects perceptions of the quality of governance more broadly (World Bank, 2020). In earlier studies about socio-economic correlates of sleep duration, economic hardship and job insecurity have been found to be related to shorter sleep duration and sleep problems (Seo et al., 2017; Salas-Nicás et al., 2020). In a large study conducted in the US, economic inequality (measured with the Gini coefficient) was associated with increased odds of inadequate and very inadequate sleep (Pabayo et al., 2022). The relationship between a country's governance quality and the national average of sleep duration has not been investigated to date.

Countries and regions differ in terms of population characteristics, which might partly explain how much the inhabitants get to sleep. In this study, the following population characteristics were investigated as correlates of sleep duration: life expectancy, median age, years of schooling, urbanization rate and the prevalence of obesity (BMI ≥ 30) among adults. The relationship between adequate sleep and life expectancy has been reported in many studies (Stenholm et al., 2019; Lu et al., 2022), although usually, sleep quality or duration has been used as a predictor of life expectancy. In the present study, we use life expectancy as a general indicator of population health and a correlate of sleep duration. Aging influences many aspects of sleep, including advanced sleep timing and shortened nocturnal sleep duration (Li et al., 2022). Consequently, countries with older populations might show different sleep duration than countries with younger populations. Schooling, urbanization, and obesity are lifestyle factors connected and possibly to sleep duration, and therefore, included in this study. However, the relationship between these variables to sleep duration is somewhat complex. For example, while urbanization with access to electricity delays sleep timing, urban populations tend to have higher sleep quality than rural populations (Beale et al., 2017). Among adults, self-reported short sleep has been found to be significantly associated with a higher incidence of obesity (Gildner et al., 2014; Wu et al., 2014; Guimarães et al., 2022). These findings underline the importance of including population variables in cross-cultural comparisons of sleep duration.

While socio-economic and population variables are likely to influence country differences in sleep duration, cultural values as the closest proxy of “culture” can be expected to be related to cultural variation in sleep. In the present study, culture was measured with Hofstede's dimensions of culture (Hofstede, 2001). Hofstede calls culture “the collective programming of the mind that distinguishes the members of one group or category of people from another” (Hofstede, 2001). The center of the mechanism of culture is “a system of societal norms consisting of the value systems (or the mental software) shared by major groups in the population.” Hofstede's cultural value model describes culture with six empirically identified dimensions. These dimensions were inequality between people (power distance), the level of stress in a society related to the unknown future (uncertainty avoidance), the integration of individuals into primary groups (individualism vs. collectivism), the division of emotional roles between men and women (masculinity vs. femininity), how the culture deals with change (long-term orientation vs. short-term orientation) and how much the society allows relatively free gratification of natural human desires related to enjoying life and having fun (Hofstede, 2001). So far, Hofstede's cultural dimensions have not been used in sleep-related studies, while we could assume that culture shapes beliefs about sleep and attitude toward sleeping. For example, we could expect societies overemphasizing success at work to prefer longer working hours and less sleep than cultures with more relaxed work attitudes (Chatzitheochari and Arber, 2009).

The aim of the present study was 2-fold. First, the aim was to investigate how socio-economic factors, population characteristics and cultural values are related to sleep duration in a set of 52 countries. Second, the relation between sleep duration and happiness (i.e., subjective well-being) was measured in the same set of countries.

## 2. Methods

The data were downloaded from various online sources. The dependent variable was sleep duration, which was adapted from Tozer (2018) based on Sleep Cycle application data. Sleep Cycle is a smartphone application (Sleep Cycle AB, Gothenburg, Sweden) that tracks night-time sound and movement. Sleep Cycle app has been used and evaluated in several studies (Patel et al., 2017; Bianchi, 2018; Choi et al., 2018; Jakowski and Stork, 2022). Countries' sleep duration ranged from 6 h 15 min (coded as score 1 for analyses) to 7 h 30 min (score 6). The other dependent variable, “national average score in happiness” (SWB), is a single-item measure in which the respondents evaluate their life with the Cantril life ladder (Helliwell et al., 2022) (see Table 1 for the item description). The independent variables included socio-economic variables (variables 3–5 in Table 1), population variables (variables 6–10 in Table 1), and Hofstede's cultural dimensions (variables 11–16 in Table 1). A description of these variables and the citations for the freely available online data can be found in Table 1. All these variables are widely used in the literature and, therefore, standardized measures of the concept that they are intended to measure. While the socio-economic variables are calculated by using national economic and social statistics, Hofstede obtained his value scores by using a standardized value survey that was administered to a large number of matched respondents in different countries, thus, providing an averaged national value score in six value dimensions and allowing country comparisons (Hofstede, 2001, 2021).

**Table 1:** Descriptions of the variables included in the study.

**Variable**	**Reference**
1. Sleep duration: time spent asleep on an average measured with Sleep Cycle application	Tozer, 2018
2. Subjective well-being (SWB): “Please imagine a ladder, with steps numbered from 0 at the bottom to 10 at the top. The top of the ladder represents the best possible life for you and the bottom of the ladder represents the worst possible life for you. On which step of the ladder would you say you personally feel you stand at this time?”	Helliwell et al., 2022
3. GDP per capita 2019: a country's average economic output per person	UNDP, 2020
4. Gini index 2010–2018: measure of economic inequality as distribution of wealth or income across a population	UNDP, 2020
5. WGI: Worldwide Governance Indicators (see the Introduction)	World Bank, 2020
6. Urbanization rate (%):the percentage of a country's population that lives in urban areas	UNDP, 2020
7. Life expectancy: Average life expectancy at birth (years)	UNDP, 2020
8. Years of schooling: the average number of years of education that a child of school entrance age can expect to receive	UNDP, 2020
9. Median age: the age that separates the higher half of a population from the lower half	UNDP, 2020
10. Prevalence of obesity (BMI ≥ 30) among adults (%): the proportion of a country's adult population that has BMI ≥ 30	World Health Organization, 2017
11. Power Distance: The extent to which the less powerful members of organizations and institutions accept and expect that power is distributed unequally.	Hofstede, 2021
12. Individualism (vs. Collectivism): The extent to which people feel independent, as opposed to being interdependent as members of larger wholes.	Hofstede, 2021
13. Masculinity (vs. Femininity): Value placed on traditionally male values: competitiveness, assertiveness, ambition, and the accumulation of wealth and material possessions.	Hofstede, 2021
14. Uncertainty Avoidance: The extent to which members of a society attempt to cope with anxiety by minimizing uncertainty; preference of rules and structured circumstances.	Hofstede, 2021
15. Short-term Orientation (vs. Long-term orientation): the extent to which a society focuses on the present and the future, vs. the past. Short-term orientated cultures are a pragmatic and adaptive approach to life while long-term oriented cultures place greater importance on the preservation of cultural values and traditions and may be less concerned with immediate material success.	Hofstede, 2021
16. Indulgence (vs. Restraint): the extent to which a society allows its members to indulge in their desires and pleasures, rather than control them.	Hofstede, 2021

The number of countries included in correlation analyses varied between 42 and 52, depending on the data availability. The data were analyzed by using Pearson product-moment correlations, partial correlations and canonical correlation analysis.

## 3. Results

### 3.1. Sleep duration and economic variables: correlation and canonical correlations

Correlations between sleep duration for each country and socio-economic indicators can be seen in Table 2. The strongest correlation was found between WGI and sleep duration. It seems that high-quality governance is positively related to a lifestyle in which long sleep is possible. In addition, income level and income inequality correlated significantly with sleep. The partial correlations (Table 2) showed that the WGI had clearly the strongest (positive) relationship with sleep duration. When the effects of all the other variables were controlled in partial correlations, the correlations between GDP and Gini index and sleep duration decreased.

**Table 2:** Correlations and partial correlations between sleep duration and socio-economic variables.

**Variable**	**1**	**2**	**3**	**Partial *R***
1. Sleep duration	1.00			
2. GDP	0.41^**^			0.11
3. Gini	−0.32^*^	−0.46^***^		−0.05
4. WGI	0.53^***^	0.80^***^	−0.46^***^	0.29

* p ≤ 0.05;

** p ≤ 0.01;

*** p ≤ 0.001.

**Figure 1:**
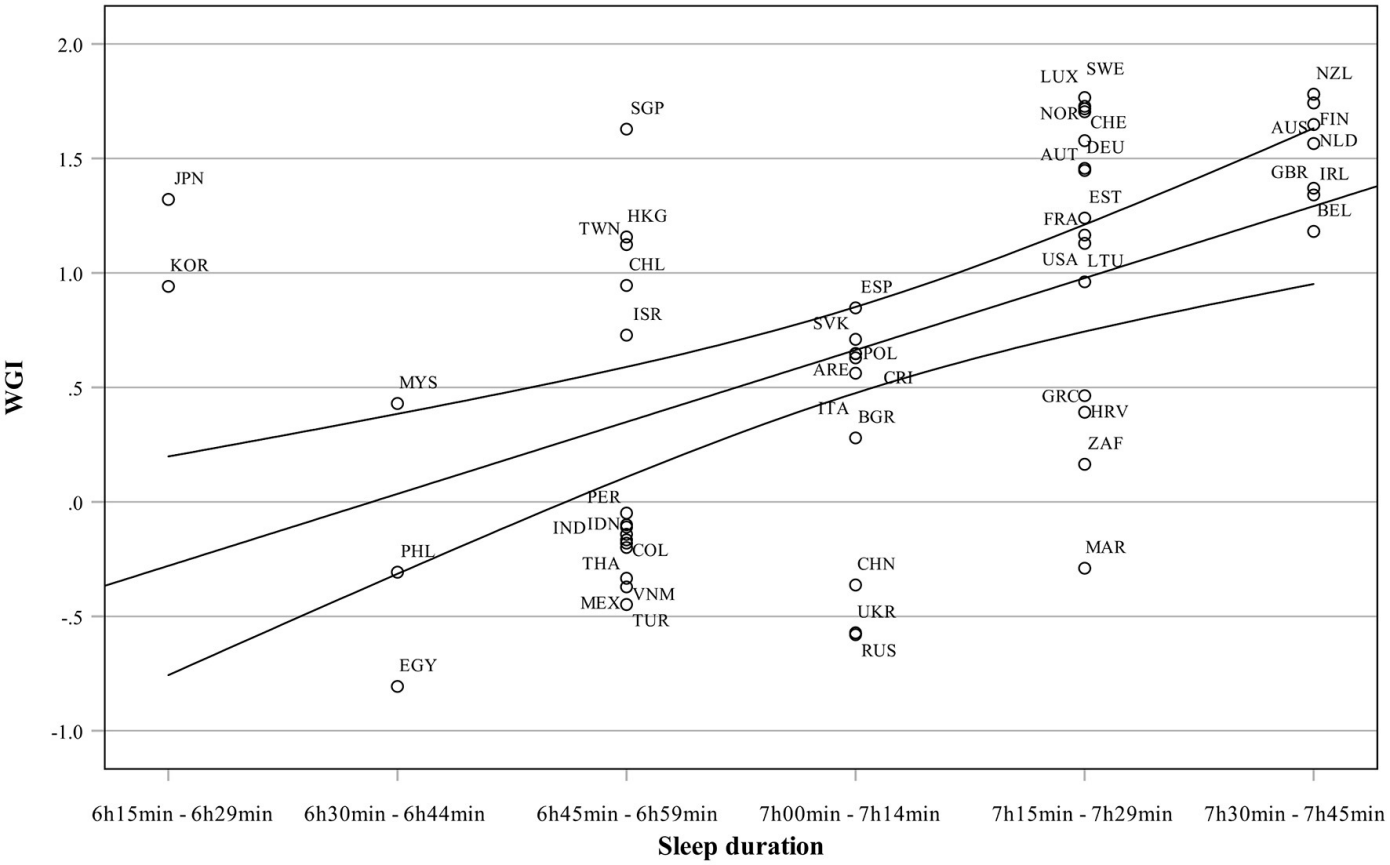
Sleep duration as a function of governance quality.

Figure 1 shows the relationship between WGI and sleep duration among the countries concerned. Countries with the longest sleep duration also score highest in governance quality. Those countries seem to be mostly welfare states. On the other end of the sleep duration continuum, countries also show lower governance quality. Outliers in this figure are Japan and Korea, which score high in WGI but very low in sleep duration.

**Figure 2:**
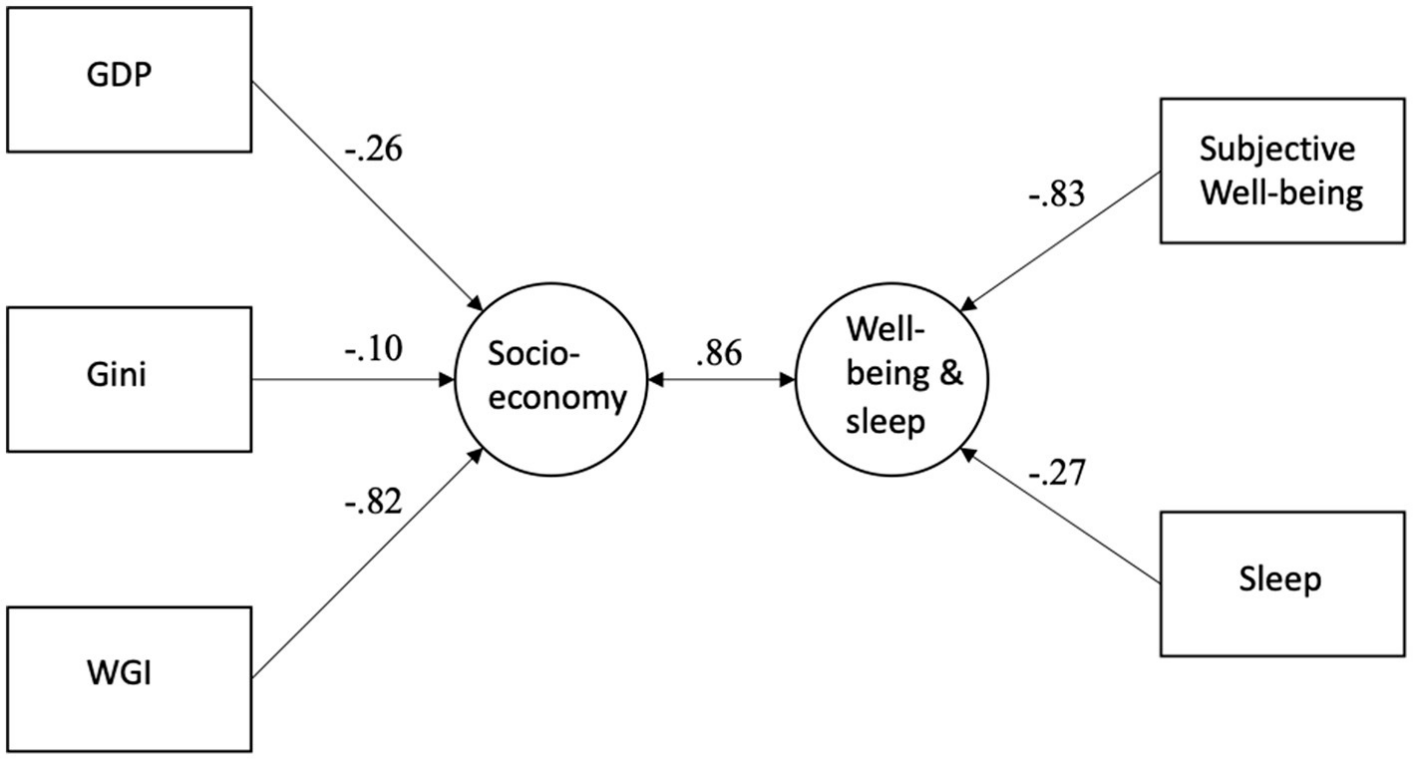
Canonical correlation analysis results for the socio-economic variables, sleep, and happiness.

To investigate how the socio-economic set of variables (GDP, Gini, WGI) is related to two well-being set (happiness and sleep), canonical correlation analysis was performed. As canonical correlation is an exploratory method and does not indicate causality, we could investigate how much the socio-economic variables (Set 2) explain of the variance in well-being variables (Set 1) and vice versa. The results can be seen in Table 3 and Figure 2. Table 3 shows that in model 1 (socio-economic model) only one canonical variate was statistically significant. The correlation between socio-economic variables (Set 2) and well-being variables (Set 1) was 0.86. The socio-economic variables (Set 2) explained 54% of variance in well-being variables (Set 1) and well-being variables explained 47% of variance in socio-economic variables. As Figure 2 shows, WGI was related to subjective well-being (happiness) and sleep.

**Table 3:** Results of the canonical correlation analysis.

**Model**	**Canonical variate**	***R***	**Eigenvalue**	**Wilks**	***F*-test (df)**
1	1	0.86	2.80	0.26	12.43^**^ (6.76)
2	1	0.80	1.77	0.20	9.68^**^ (4.40)
	2	0.67	0.81	0.55	8.11^**^ (4.40)
3	1	0.84	2.31	0.20	6.20^**^ (12.60)
	2	0.58	0.52	0.66	3.21^*^ (5.31)

* p ≤ 0.05;

** p ≤ 0.001.

### 3.2. Sleep duration and population variables: zero-order correlations and canonical correlations

Table 4 presents correlations and partial correlations between population variables and sleep duration. The most significant correlates of sleep duration were the amount of schooling and obesity percentage in the population. Life expectancy and median age correlated with sleep duration, but the correlations were clearly weaker. In partial correlations, the obesity and schooling had the strongest correlations with sleep.

**Table 4:** Correlations and partial correlations between sleep duration and population variables.

**Variable**	**1**	**2**	**3**	**4**	**5**	**Partial** ***R***
1. Sleep duration	1.00					
2. Life expectancy	0.28^*^					−0.09
3. Median age	0.35^*^	0.64^***^				0.28
4. Years of schooling	0.57^***^	0.67^***^	0.46^***^			0.43
5. Urbanization rate	0.17	0.60^***^	0.31^*^	0.60^***^		−0.27
6. Obesity (%)	0.55^***^	0.14	0.09	0.42^**^	0.35^*^	0.48

* p ≤ 0.05;

** p ≤ 0.01;

*** p ≤ 0.001.

**Figure 3:**
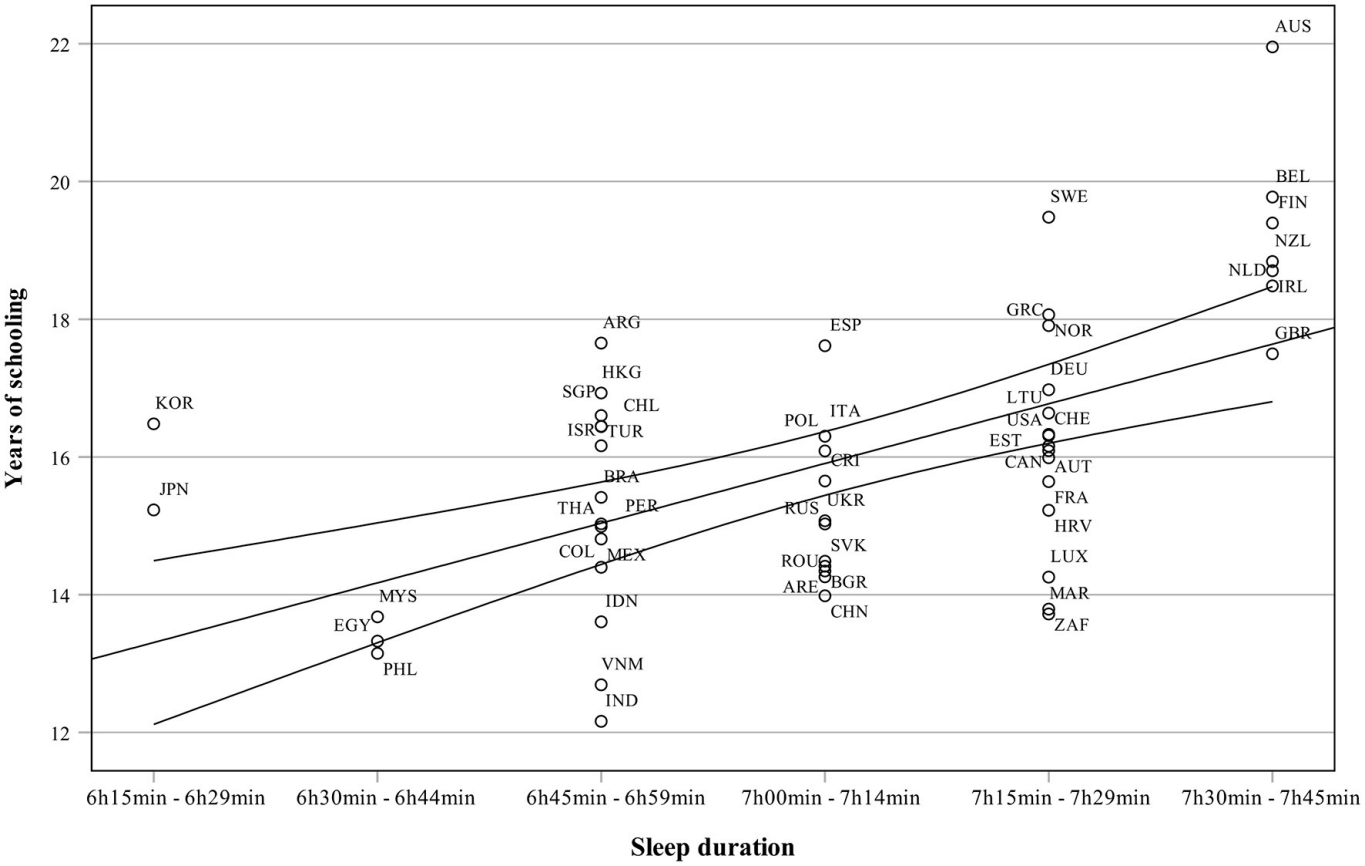
Sleep duration as a function of years of schooling.

**Figure 4:**
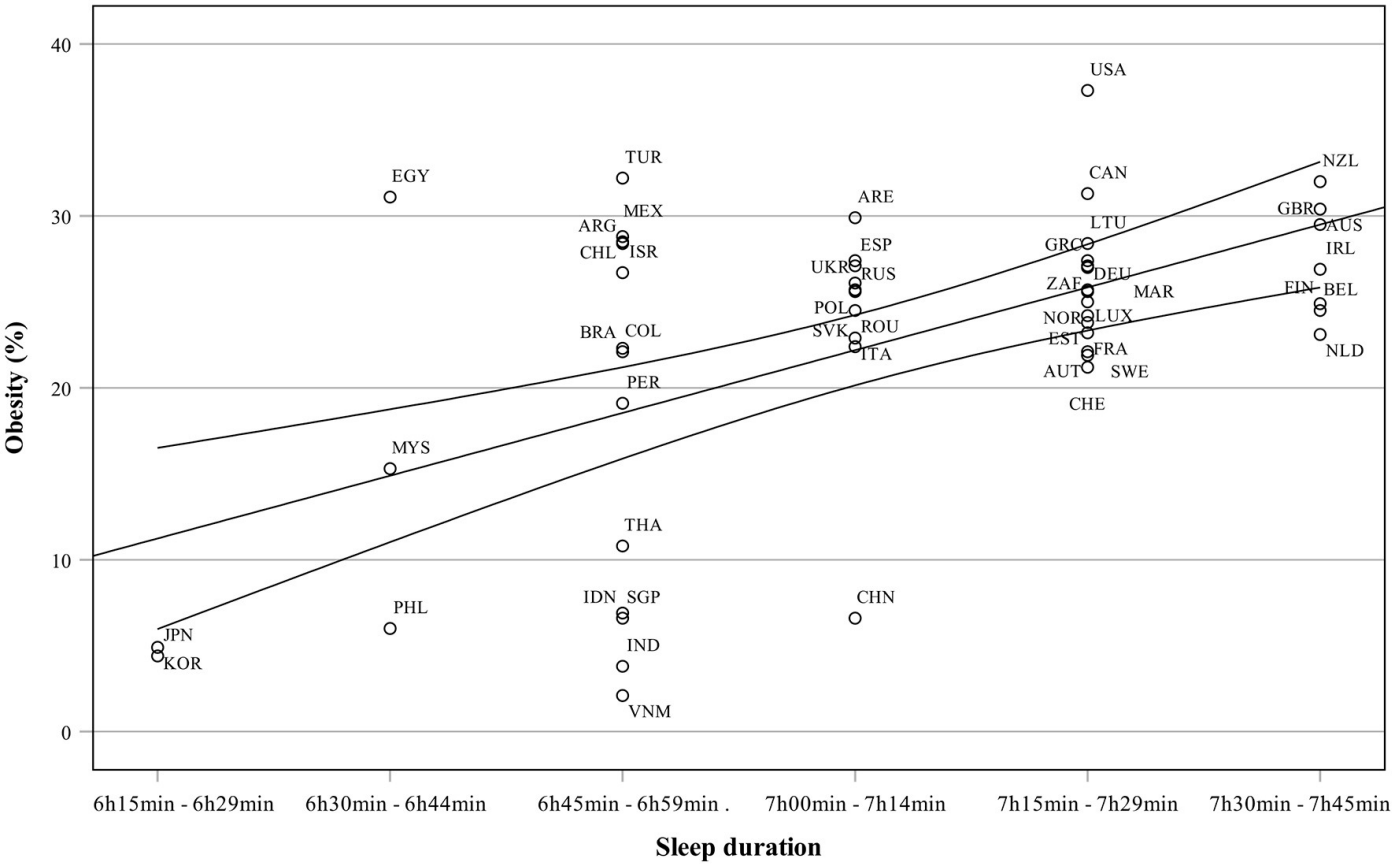
Sleep duration as a function of obesity.

Figure 3 presents sleep duration as a function of years of schooling. Countries with the longest schooling and highest sleep duration are Australia, Belgium, New Zealand, and Finland, while Malesia, Egypt and the Philippines have both the shortest sleep duration and schooling. The relationship between obesity and sleep is presented in Figure 4. Many highly industrialized countries such as New Zealand and the UK have both high sleep duration and obesity percentages. Japan and South Korea are examples of countries with low obesity rates but also low sleep duration.

**Figure 5:**
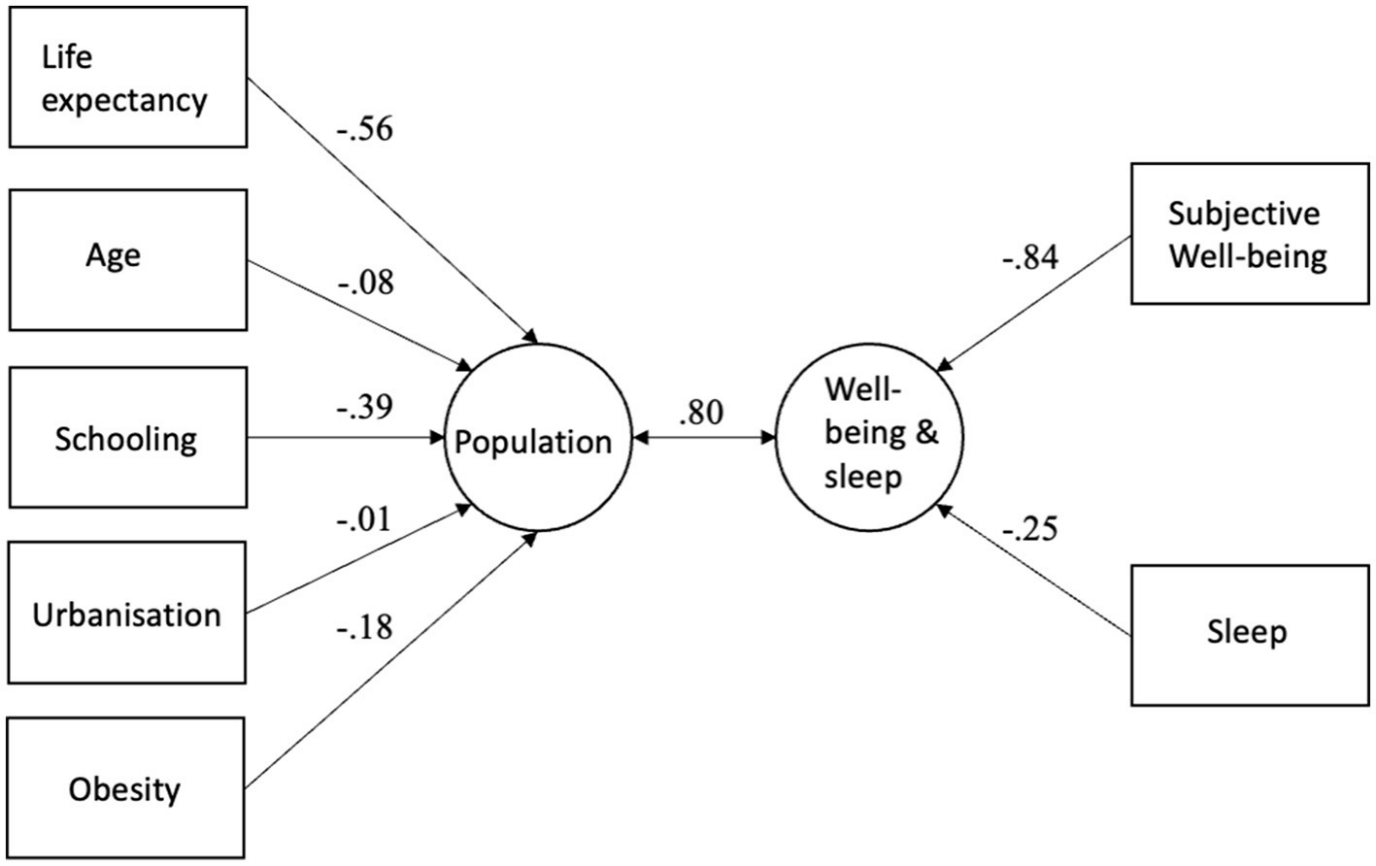
Canonical correlation analysis results for the population variables, sleep, and happiness.

The canonical correlation analysis yielded in two significant canonical variates (Table 3). Since the eigenvalue of the second canonical variate was very low (0.81), results related to the first canonical variate are presented here. The results can be seen in Table 3 and Figure 5. The correlation between population variables (Set 2) and well-being variables (Set 1) was 0.80. The population variables (Set 2) explained 47% of variance in well-being variables (Set 1) and well-being variables explained 34% of variance in population variables.

### 3.3. Sleep duration and cultural values: correlation and regression analyses

**Figure 6:**
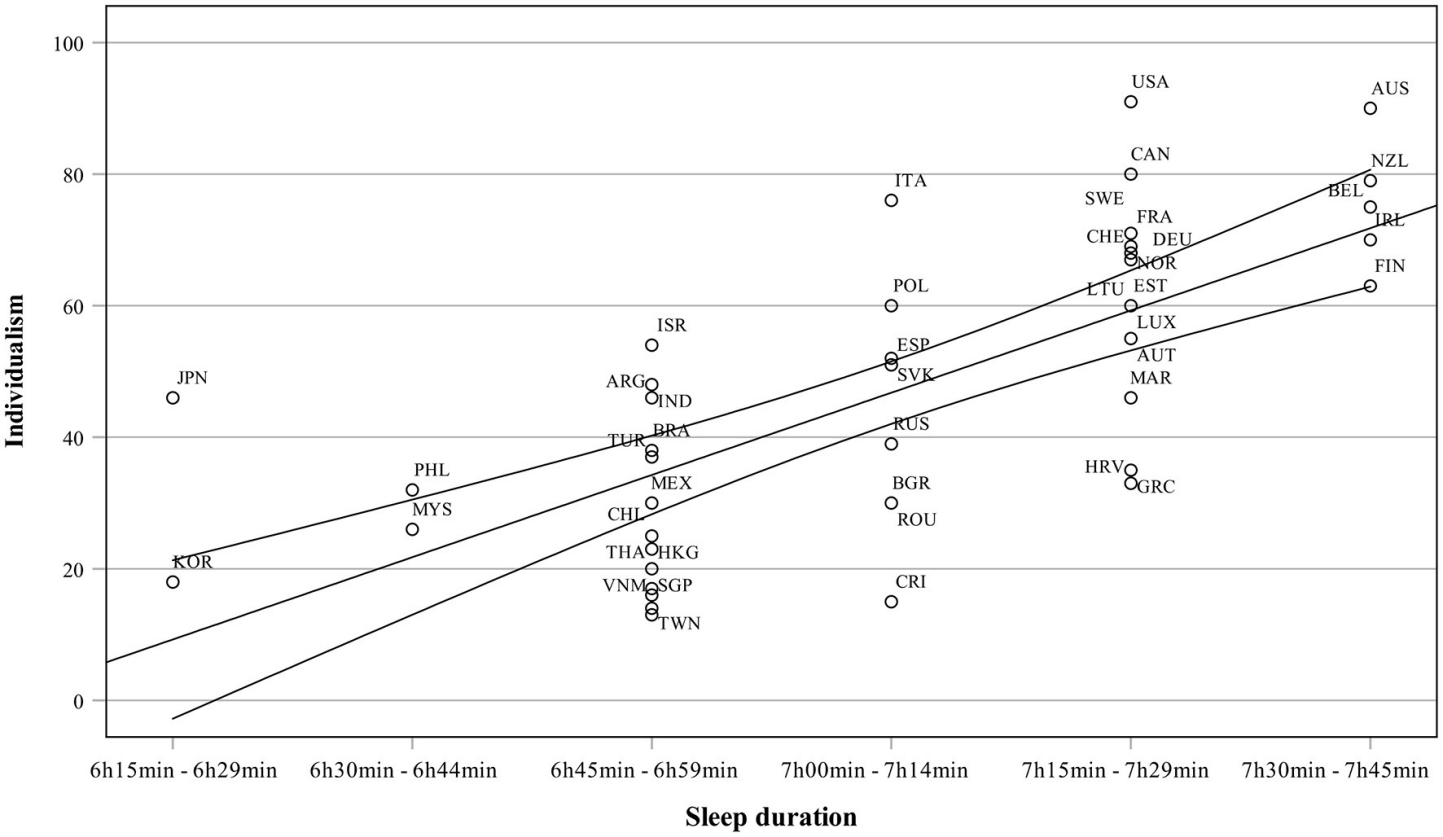
Sleep duration as a function of individualism.

Table 5 presents the correlations between Hofstede's cultural dimensions and sleep duration. Only power distance and individualism correlated statistically significantly with sleep duration. While sleep duration was positively related to individualism, power distance correlated negatively with sleep duration. It should be noted that individualism and power distance are strongly interrelated (*r* = −0.55). In partial correlations, strongest relations to sleep duration were found for individualism (0.62) and masculinity (−0.26). The relationship between sleep duration and individualism is presented in Figure 6, which shows a strong connection between individualism and sleep duration. Countries scoring high in sleep duration score at least moderately in individualism. South Korea is an example of the other end, low individualism, and low sleep duration.

**Table 5:** Correlations and partial correlations between sleep duration and cultural values.

**Variable**	**1**	**2**	**3**	**4**	**5**	**6**	**Partial *R***
1. Sleep duration	1.00						
2. Power distance	−0.50^***^						−0.17
3. Individualism	0.71^***^	−0.55^***^					0.62
4. Masculinity	−0.12	0.16	0.15				−0.26
5. Uncertainty avoidance	−0.07	0.06	−0.14	0.02			0.08
6. Long-term orientation	−0.05	0.07	−0.06	0.02	0.08		−0.15
7. Indulgence	0.24	−0.37^*^	0.23	0.09	−0.09	−0.41^**^	−0.10

* p ≤ 0.05;

** p ≤ 0.01;

*** p ≤ 0.001.

**Figure 7:**
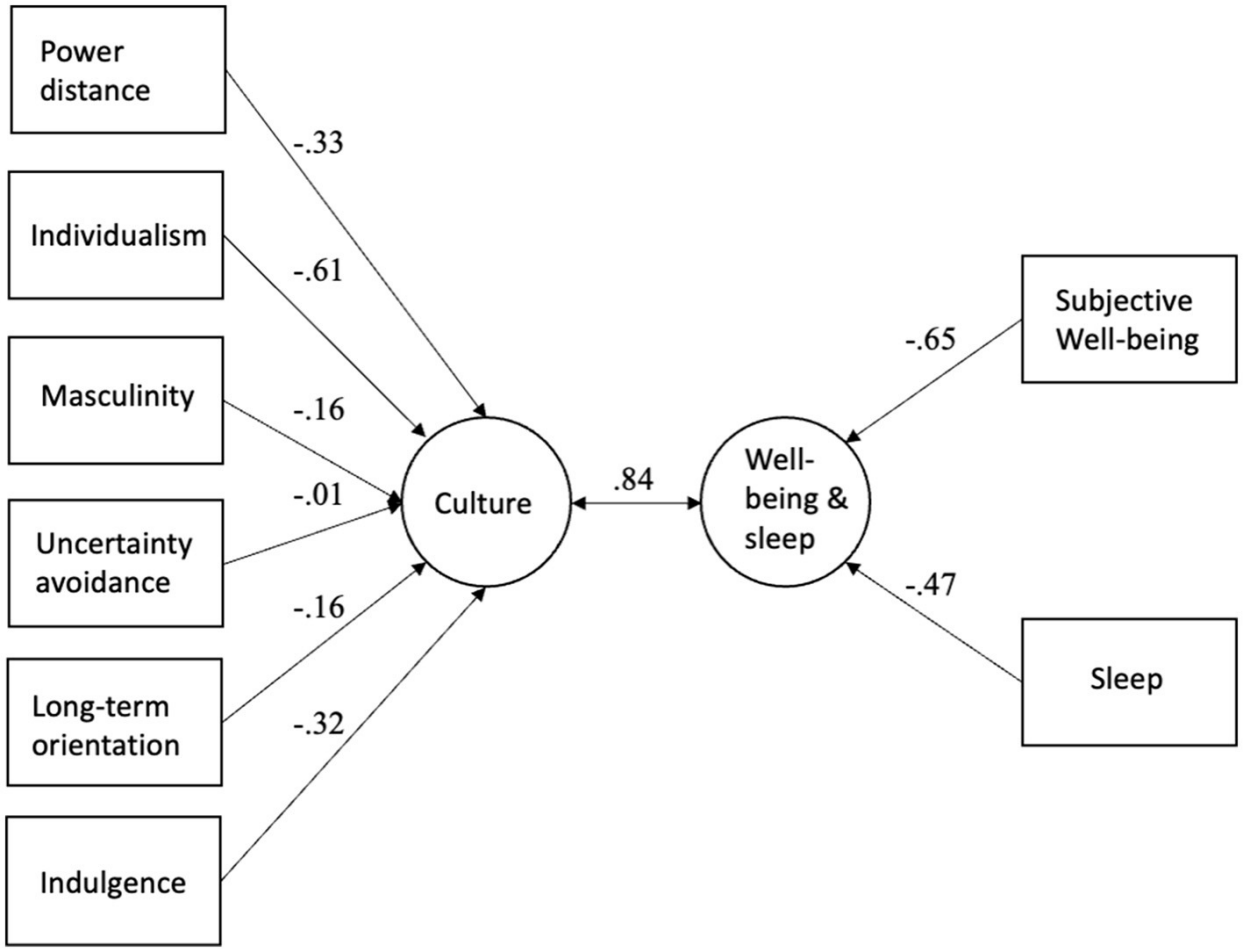
Canonical correlation analysis results for the cultural values, sleep, and happiness.

Two canonical variates were statistically significant in the canonical correlation analysis of well-being variables (Set 1: sleep and happiness) and cultural values (Set 2: Hofstede value dimensions). As Table 3 shows, eigenvalue of the second variate was low and, thus, detailed results are presented here only for the first canonical variate. The results can be seen in Figure 7. The correlation between cultural values (Set 2) and well-being variables (Set 1) was 0.84. The cultural values (Set 2) explained 56% of variance in well-being variables (Set 1) and well-being variables explained 20% of variance in cultural values. All these analyses show that the sleep duration and happiness are most strongly related to individualism.

### 3.4. Sleep duration and subjective well-being: correlation analysis

The study's second aim was to investigate the relationship between sleep duration and happiness (i.e., subjective well-being). Correlation coefficients between happiness, sleep duration, socio-economic variables, population variables and cultural values are presented in Table 6. Table 6 shows a relatively strong relationship (*r* = 0.54, *p* < 0.001) between sleep duration and happiness.

**Table 6:** Correlations between subjective well-being, sleep duration, and independent variables.

**Variable**	***R***
Sleep duration	0.54^**^
GDP per capita	0.68^**^
Gini index	−0.32^*^
WGI	0.82^**^
Life expectancy	0.76^**^
Median age	0.46^**^
Years of schooling	0.66^**^
Urbanization rate (%)	0.55^**^
Prevalence of obesity	0.29
Power distance	−0.68^**^
Individualism	0.56^**^
Masculinity	−0.09
Uncertainty	−0.13
Long-term orientation	0.13
Indulgence	0.52^**^

* p ≤ 0.05;

** p ≤ 0.001.

Table 6 shows that happiness was positively related to governance quality (WGI), urbanization, life expectancy, years of schooling, median age, Individualism, and indulgence and negatively with inequality and power distance. Hence, the relationship between sleep duration and happiness was about at the same level as between urbanization rate, individualism, and indulgence, which may underline the importance of sleep duration in subjective well-being.

**Figure 8:**
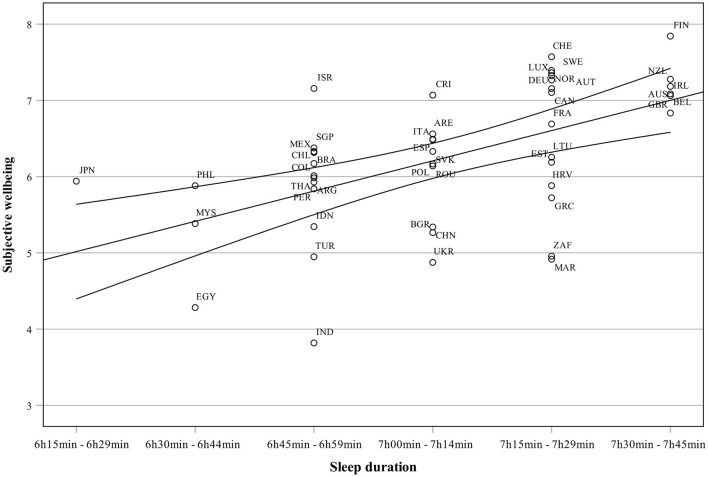
Subjective well-being (SWB) as a function of sleep duration.

The relationship between sleep duration and subjective well-being is shown in Figure 8. Countries scoring highest in sleep duration (Finland, New Zealand, Ireland) scored highest also in subjective well-being. Likewise, countries with the lowest subjective well-being score (Egypt, India, Turkey) scored relatively low (but not lowest) in sleep duration too. Japan is an interesting outlier: while having the shortest sleep duration, the Japanese score relatively high in subjective well-being. In general, however, longer sleep duration is related to subjective well-being and happiness.

## 4. Discussion

A widely accepted consensus seems to be that sleep is one of the key factors affecting health and well-being (St-Onge et al., 2016). Both too little sleep and an excessive amount of sleep are related to a multitude of illnesses, such as cardiovascular diseases, cancer, metabolic syndrome, and stroke (Lee et al., 2015; Sivertsen et al., 2021; Li et al., 2022). International data show that there are significant regional and cultural differences in sleep duration (Tozer, 2018). People getting the least amount of sleep seem to be mainly in Asia and the Middle East, while the Scandinavians, together with Australians and New Zealanders, seem to get the longest sleep. The present study aimed to investigate how socio-economic factors (GDP per capita, quality of governance, income equality), population characteristics (education, obesity, aging, life expectancy, urbanization) and cultural value dimensions (Hofstede's cultural value dimensions) are related to sleep duration.

The most important and finally only significant socio-economic factor was governance quality (WGI). The WGI is an aggregate score of six dimensions of governance: Voice and Accountability, Political Stability and Absence of Violence, Government Effectiveness, Regulatory Quality, Rule of Law, and Control of Corruption (World Bank, 2020). In the present study, WGI had a strong positive correlation to the country's income level (GDP per capita) and a moderate negative correlation to income inequality (Gini index). Good governance with high predictability and job security seems to reduce people's worries about their future need for extra income from second jobs and overwork. While WGI has not been used before in sleep research, earlier studies confirm the importance of job security and economic stability in sleep quality (Seo et al., 2017; Salas-Nicás et al., 2020; Pabayo et al., 2022).

Many chronic illnesses such as diabetes mellitus (Type 2), cardiovascular diseases and cancer are more prevalent in countries characterized by certain lifestyle characteristics such as unhealthy diets, sedentary lifestyles and widespread use of alcohol and tobacco. Urban lifestyles with hectic work schedules and a vast number of stimuli such as social media and entertainment compromise the hours spent in sleep both among adults and children (Bapat et al., 2017). In the present study, especially years of schooling and obesity had strong correlations with sleep, while in partial correlations also, urbanization rate appeared as an important correlate. The proportion of obese people in the population and average years of schooling were positively and urbanization negatively related to sleep duration. The positive relationship between education and sleep, as well as the negative relationship between urbanization and sleep duration, are easy to understand. However, the positive relationship between obesity and sleep duration seems to contradict previous studies in which short sleep duration has been related to obesity (Gildner et al., 2014; Grandner et al., 2015; Guimarães et al., 2022). It should be noted, however, that in previous studies, the focus has been on the effects of short sleep duration on overweight, not vice versa. Moreover, the relationship between sleep and obesity seems to be complex, changing as a function of age (Grandner et al., 2015). More research at the country level is needed to clarify the direction and shape of this relationship.

In the present study, cultural values were measured with Hofstede's widely used model of cultural value dimensions (Hofstede, 2001, 2021). Especially individualism but also masculinity in partial correlations were related to sleep duration. Sleep duration was also related (negatively) to power distance, but in partial correlations, the relationship between sleep and power distance diminished significantly due to a strong correlation between individualism and power distance. Sleep duration was positively related to individualism (partial *r* = 0.62) and negatively to masculinity (partial *r* = −0.26). Masculine cultures are characterized by such values as being successful and competitive, especially in work life. Overemphasis on work might lead to overworking and work stress, which can influence sleeping habits. Positive correlations between sleep duration and individualism, even when controlling for GDP, might be related to an individual's autonomy, which is the defining characteristic of individualism. Hofstede (2021) defines individualism as “the extent to which people feel independent, as opposed to being interdependent as members of larger wholes.” In individualistic countries, people are allowed and even expected to define their own lifestyle instead of following the customs of the community. Hence, individualism gives a person a chance to choose his or her sleeping habits, which might fit better to a person's chronotype, i.e., being a “night owl” or “early bird,” which might reduce the adverse effects of the mismatch between chronotype and requirements of the work-life (Partonen, 2015). While cultural values seem to influence how much people sleep, the role of cultural values in sleep health should not be exaggerated. Such factors as lack of safe, clean sleeping spaces, overcrowding, and other disturbing factors (e.g., noise, air and light pollution) are important factors affecting an individual's sleep health, especially among marginalized such as homeless people regardless of their cultural background. On the other hand, the dominant culture in a country can either promote decent living conditions for marginalized people or exacerbate social problems, including low sleep health.

The results discussed above were related to the national factors related to sleep duration. While knowing the societal factors facilitating a good night's sleep are highly relevant for planning the societal and work life, it is even more important to investigate the relationships between sleep duration and health, especially mental health. It has been reported in many studies that low sleep quality or duration has negative effects on self-evaluated happiness, well-being, and life satisfaction (Shin and Kim, 2018; Zhao et al., 2019; Otsuka et al., 2020; Kouros et al., 2022; Kukade et al., 2022). In the present study, the relationship between mental health and well-being was studied by correlating sleep duration with subjective well-being (happiness) measured with the Cantril life ladder (Helliwell et al., 2022). The average sleep duration explained 30% of the variance in happiness. Taken that the sleep duration is a very rough measure of sleep quality (or actually quantity), the strength of the relationship between sleep duration and happiness is impressive in the present data. While adequate sleep can be assumed to increase the prevalence of positive emotions and subjective well-being, also subjective well-being and experienced happiness can be expected to increase the likelihood of good sleep. An individual experiencing stress, worry and hardship in his or her life is less likely to enjoy a good sleep than an individual with only a few stressors in life. Similarly, sleeping enough is essential for mental well-being and happiness. The results of the present study underline the relationship between sleeping well and subjective well-being.

The sleep duration and subjective well-being (happiness) were combined in canonical correlation analysis as one “well-being set.” The canonical correlations between this “well-being set” and other sets of variables (i.e., socio-economical set, population characteristics set, cultural values set) were calculated, and the amount of variance explained by the different sets analyzed. The analyses show that the well-being set, including sleep and happiness, correlated strongly with the socio-economic set (*r* = 0.86), population set (*r* = 0.80) and cultural values set (*r* = 0.84). In every model, the other sets explained more variance in well-being set than vice versa, which might give some hints about the direction of the relationships. Since the data could be considered cross-sectional, it is not important not to draw far-reaching, if any, conclusions based on causality.

This study has some limitations which must be considered when evaluating the applicability of the results. First, the study design is ecological, i.e., the cross-sectional country-level data were analyzed by using correlations and canonical correlations. Thus, the unit of observation was not a person or a patient but an entire population in a country. While this kind of design using aggregated values can reveal important macro-level relationships between socio-economic variables, sleep, and health outcomes, the detailed physiological mechanisms between these variables cannot be investigated. It is also important to understand that findings at a group level or at the level of society do not allow conclusions at an individual person's level. While a group is formed by individuals, group-level statistics apply only to groups and not to individual people. For example, a person having a shorter sleep duration than recommended can be as happy as a person sleeping longer. The results indicate, however, that countries in which people sleep less score lower in happiness (or vice versa).

Second, correlational design and cross-sectional regression analyses cannot demonstrate causal relationships. While it is reasonable to assume that relatively stable cultural factors or economic outcomes such as GDP or income equality influence people's possibilities of getting enough sleep, also the opposite direction of the relationship is possible, at least in theory. It should be noted, however, that socio-economic and cultural factors are multi-faceted and very durable, while sleep duration is just one single measure. Change in cultural or population characteristics is very slow and almost impossible to induce by any interventions, whereas changing the sleep duration should be much easier. For example, later start times for school and work, flexible and shorter work hours and controlling the bedtime of children and teenagers, including the ban on late evening smartphone use, can drastically change the sleep duration of adults and adolescents. While this study was strictly speaking cross-sectional by nature (despite the fact that many socio-cultural variables were recorded earlier than the sleep duration), we may assume that socio-cultural variables are more likely to influence sleep duration and happiness than vice versa, which was also partly demonstrated in canonical correlation analyses. We can conclude that more studies with longitudinal (time-series) design are needed to reveal the direction of the relationship between socio-economic factors, sleep duration, and happiness.

Third, this study utilized sleep duration as an index of good sleep. It should be noted, however, that sleep duration is a crude measure, not a comprehensive measure of the restorative nature of sleep. While too short (or long) sleep is a good measure of inadequate sleep, sleep duration should be accompanied by measures of sleep quality. Unfortunately, such comprehensive measures of sleep quality are much more difficult to obtain for a large set of countries than the crude sleep duration. Sleep duration is easy to measure with smartphone applications such as the Sleep Cycle app and wearable (e.g., wrist or finger-worn) devices or with a simple survey, while measurement of sleep quality with these commercial applications and self-report surveys is more or less inaccurate (Ameen et al., 2019). The sleep duration measured with smartphone applications or trackers is a crude estimate, which should be interpreted with caution. It should be noted that this is the case with all country-level macro-indicators, whether they measure economy or population characteristics. Sleep trackers, however, are valuable for collecting huge amounts of data from many countries simultaneously, which would be otherwise impossible to obtain (Ameen et al., 2019).

Fourth, compared to experimental designs, ecological design is always vulnerable to confounding variables, which might, in the worst-case, lead to misinterpretation of the results. In the case of the present study, an unidentified variable could be related to both sleep duration and happiness and, thus, cause a spurious correlation between these two variables. While the complete exclusion of this kind of bias is never possible in correlative studies, we tried to include in the study all the relevant socio-economic, population and cultural variables, which could be assumed to have a relationship between sleep duration and subjective well-being. The partial correlation and canonical correlation analyses should reveal the relationships between individual variables and sets of variables and, in this way, reduce the possibility of confounding variables biasing the results.

Finally, this study utilized Hofstede's cultural value dimensions as an index of national culture. While Hofstede's dimensions have been used in numerous studies and, thus, can be considered reliable and valid measures of culture, reducing “culture” to six dimensions is naturally an oversimplification. Moreover, Hofstede's dimensions measure “dominant” or “majority” culture ignoring cultural diversity within a country. Different ethnic and cultural minorities may have different lifestyles, which might also be reflected in their sleep patterns. On the other hand, Hofstede's dimensions as an index for the majority culture are likely to influence all inhabitants in a country because the functioning of the society is organized based mainly on the majority's values.

## 5. Conclusion

The present study shows that socio-economic factors, population characteristics and cultural values are related to sleep duration at the country level. The national average of sleep duration correlated with happiness (subjective well-being), which indicates either that people in countries with longer sleep duration are happier or that in happy countries people sleep longer. Whatever the direction of the relationship is, sleep and happiness are strongly related to each other. Countries aiming at improving well-being and happiness at the population level should aim at improving conditions for sleep. The political interventions for improving sleep quality could include arrangements related to work life (e.g., flexible and shorter working hours) and school (e.g., later starting time for the school day), as well as a clearer separation between work and free time. When designing interventions for better sleep, the cultural characteristics of the country should be considered. In an individualistic country, the benefits of good sleep for an individual should be emphasized, while in collectivistic countries, a more collectivistic approach underlining, for example, the importance of good sleep for family life is needed.

When assessing the applicability of the findings, it is essential to understand that the present study was an exploratory study about socio-economic and cultural aspects of sleep at the country level and, therefore, cannot be applied at the level of individuals or even groups of people. Future confirmatory studies are needed to establish the causal relationship between sleep, happiness and societal factors before further conclusions can be drawn. The present study is an opening for a new line of research in which sleep is seen as an essential part of societal life and collective well-being.

## Data availability statement

Publicly available datasets were analyzed in this study. This data can be found at: references to open access data sources can be found in Table 1.

## Author contributions

TL conducted the data analyses and prepared the first draft of the manuscript. All authors contributed to the conceptualization and design of the study, the acquisition and interpretation of the data, and conceptualization of the manuscript, as well as editing and approving the submitted version.
